# Sex differences in intuitive eating and its relationship with body mass index among adults aged 18–40 years in Saudi Arabia: a cross-sectional study

**DOI:** 10.3389/fnut.2023.1214480

**Published:** 2023-07-19

**Authors:** Eram Albajri, Manal Naseeb

**Affiliations:** Clinical Nutrition Department, Faculty of Applied Medical Sciences, King Abdulaziz University, Jeddah, Saudi Arabia

**Keywords:** intuitive eating, IES-2, Saudi Arabia, body mass index, obesity, sex

## Abstract

**Introduction:**

Intuitive eating (IE) is eating without judgment, relying only on physiological hunger and satiety. Sex differences in IE have been reported; however, none of the studies have explicitly examined IE and its relationship with body mass index (BMI) in the Saudi Arabian population. Thus, this study aimed to investigate sex differences in IE and its relationship with BMI in the Saudi population.

**Subjects/methods:**

A cross-sectional online survey of 360 participants (18 years or older) with self-reported weight and height was conducted. IE was measured using the Intuitive Eating Scale-2 (IES-2). Separate multiple linear regression analyses were conducted to determine if total IE and its subscale scores differed across sexes. It was also conducted to assess the relationship between IE and BMI across sexes.

**Results:**

Women had higher total IE score, eating for physical rather than emotional reasons (EPR), and body-food choice congruence (BFCC) scores compared to men (*p* = 0.013, *p* = 0.01, *p* <0.001, respectively). The analysis showed a significant negative association between total IE, BFCC, EPR scores, and BMI in women compared to men (*p* = 0.023, *p* = 0.01, *p* = 0.003, respectively).

**Conclusion:**

The data on the sex differences in IE and its subscales and their different association with BMI encourage tailing nutrition-related recommendations in the context of intuitive eating based on sexes. Future studies are needed to explore how intuitive eating functions differently in women compared to men and explore the causal relationship between IE and BMI in this population.

## 1. Introduction

Globally, the prevalence of obesity has increased. Saudi Arabia has a higher rate of overweight individuals and obesity, with three out of five adults being overweight or obese in the year 2019 (1). Obesity is a complex disease associated with mental problems and several chronic diseases that are known as the leading cause of death worldwide such as type 2 diabetes, cardiovascular diseases, and some cancers (2, 3). In the Middle East, Saudi Arabia has the second highest rate of diabetes and ranks seventh worldwide (4). It is noteworthy that this disease is primarily linked to obesity and can be managed by preventing weight gain and obesity. The adverse impact of obesity on health as well as the alarming rate of increased prevalence indicate the need for more effective prevention and treatment strategies.

Traditional weight loss approaches, such as food restriction or high-intensity exercises, might be unsuccessful for long-term weight loss and could be physically and mentally detrimental to the individual (5). Studies have shown that long-term food restriction may lead to disturbed eating behavior and improper food and body relationships (6). In addition, this may result in repeated cycles of weight loss and regain, a reduction in self-trust with food, and eventually obesity or eating disorders (7). The effectiveness of long-term dieting on weight loss and healthy lifestyle is limited; therefore, an alternative to traditional restrictive eating is needed to reduce these disordered eating attitudes and related psychological problems.

A growing body of research supports the anti-dieting approach, which involves recognizing and reacting to individuals’ signals of hunger and satiety. There has been a shift in the dieting approach, and it now focusses on connecting with the body and trusting its need to adopt a healthy lifestyle (8, 9). One of the approaches that applies this concept is known as Intuitive eating (IE). In 1995, clinical dietitians Evelyn Tribole and Elyse Resch identified and suggested IE as an off-diet approach for unsuccessful long-term weight loss. IE is an eating behavior which is guided by physiological hunger and satiety sensations (i.e., eating when hungry and stopping when full). The principles of IE encouraged a healthy relationship that connect food, mind, and body. They also promote an appreciation of emotions and enjoyment of food. Therefore, it allows individuals to trust their body to make choices around nutritious and energetic food; and gives a sense of pleasure without the effect of emotional or external cues, such as altered mood (10, 11).

IE is measured by the Intuitive Eating Scale-2 (IES-2) (12). IES-2 includes four subscales which reflect the essential aspects of IE. “Unconditional permission to eat (UPE)” subscale assesses the act of eating whatever food is desired at the moment. “Eating for Physical Rather Than Emotional Reasons” (EPR) subscale assesses the act of eating that is governed by energy needs rather than eating that is driven by emotions. “Reliance on Hunger and Satiety Cues (RHSC)” subscale assesses individuals’ ability to tune into their body’s hunger and satiety signals in relation to food intake. “Body-Food Choice Congruence (B-FCC)” subscale assesses the choice of tasty and healthy nutrition in line with the bodily needs (12, 13). Combining the scores of these aspects provides a total IE score that reflects whether individuals are intuitively eating.

The IES-2 scale has been used in both female and male populations and has demonstrated invariance across sexes. This means that scores can be compared and interpreted across sexes. Interestingly, researchers have shown inconclusive results when comparing the scores of men to women as they might be equal, higher, or lower (12, 14–18). Furthermore, it has also been demonstrated that women and men may have different associations with IES subscales (15, 19). Several factors could have contributed to this sex discrepancy, such as cross-cultural differences in food. Understanding the interaction between sex and IE will aid in understanding how IE works for both men and women. Therefore, examining sex differences in IE practices across different cultures is imperative.

Research on IE holds promise for improving weight management, physiological and psychological health, dietary quality, as well as quality of life (7, 20, 21). Clinical studies have indicated that IE is related to weight stability as opposed to other weight loss diets approaches (20, 22). Researchers examining the IE approach found a relationship between IE and anthropometric measurements (12, 16, 18, 19, 23). The higher the IE, the lower was the Body Mass Index (BMI), body weight, waist circumference, and waist-to-hip ratio (23). Research on early adolescents, young adults, college students, and adults have also shown a negative relationship between IE and BMI (12, 16, 18, 19). Moreover, a mounting body of evidence indicates that intuitive eaters have lower BMI and higher life satisfaction and self-esteem (5, 12, 18, 24, 25). Previous studies have found that EPR and RHSC are negatively correlated with BMI; however, the findings are inconsistent for UPE and B-FCC (12, 19, 26). Therefore, it is important to examine the relationship between IE, and its subscales and BMI as a potential approach to compete with obesity and promote weight maintenance (27, 28).

While existing research on IE is promising, little is known about IE practices and their relationship with BMI across sexes in Saudi Arabia. Therefore, the current study aimed to (1) examine sex differences in IE practices using IES-2 in Saudi Arabian population and (2) assess the relationship between IE and its subscales with BMI based on sexes within the target population. This might contribute to the development of alternatives to the current strategies of obesity management programs used in Saudi Arabia. Consequently, the prevalence of obesity, diabetes, and other diseases would also decrease. Based on prior findings, we hypothesized that women would have lower IE scores compared to men. Additionally, we also hypothesized a significant negative relationship between IE and BMI.

## 2. Materials and methods

### 2.1. Participants and procedures

For this cross-sectional study, we aimed to recruit at least 360 participants (1:1.3 ratio; men to women). The potential sample size was determined using a two-sided *t*-test, with confidence level of 95%, power of 80%, and estimated effect size 0.3. Data was collected from October 2020 to August 2021. An anonymous online survey was created and circulated among community members via social media (such as twitter) and word of mouth. Before answering the questionnaire, the survey presented information on the study objectives, protocol, confidentiality statements, and consent statements. Sociodemographic characteristics, anthropometric measurements, and the IES-2 were included as part of the questionnaire.

A convenient sample of 360 participants was included in the study, of which 153 were men (42.5%) and 207 women (57.5%). Adults between the ages of 18 and 40 years of either sex with a BMI of 18.5 or above, who were fluent in English and Arabic, and were living in Saudi Arabia were included in the study. The exclusion criteria included history of bariatric surgery or surgery within the next 12 months; history or current eating disorder; history of metabolic or mental health disorders; use of prescription medications that affect eating or metabolism, pregnancy, or breastfeeding within the last 12 months; attempt to lose more than 4.5 kilograms during the last 3 months; food allergies or special diets; and individuals residing outside Saudi Arabia. Approval to conduct the study was obtained from the Research and Ethics Committee of the Faculty of Applied Medical Sciences of King Abdulaziz University (reference no. FAMS-EC2020-0016).

### 2.2. Measures

#### 2.2.1. Sociodemographic characteristics

Data regarding participants’ sex, age, region of residence, education status, employment status, socioeconomic status, and weight loss dieting practices was collected via the questionnaire. Participants reported their current weight and height which were used to calculate their body mass (kg/m^2^). World Health Organization classification of BMI was used in this study (4).

#### 2.2.2. Intuitive eating scale (IES-2)

IES-2 was used to assess intuitive eating (IE). The IES-2 consists of 23 items and four subscales. The subscales included: (1) ‘Unconditional Permission to Eat’ (UPE; six items, e.g., “If I am craving a certain food, I allow myself to have it.”); (2) ‘Eating for Physical Reasons Rather than Emotional Reasons’ (EPR; eight items, e.g., “I am able to cope with my negative emotions (e.g., anxiety, sadness) without turning to food for comfort.”); (3) ‘Reliance on Hunger and Satiety Cues’ (RHSC; six items, e.g., “I rely on my hunger signals to tell me when to eat.”); (4) ‘Body-Food Choice Congruence’ (B-FCC; three items, e.g., “I mostly eat foods that give my body energy and stamina.”). Participants were asked to rate each item on the scale using a 5-point Likert scale for scoring ranging from one (strongly disagree) to five (strongly agree). The IES-2 yields separate scores for each subscale as well as a composite total score. After reversed scoring items in the scale, the total score was created by summing the results of each item and dividing them by 23 for a total average score (12). Similarly, subscales scores were calculated by summing up the responses and dividing them by the number of items for each subscale individually. Possible scores on the scale range from 1 to 5 with higher scores indicating greater intuitive eating. Previous research has supported the validity and reliability of the total IES-2 and its subscales scores, as it showed good internal consistency, test–retest reliability, and construct validity (12, 15). Moreover, it has also been applied to cross-cultural samples and shown to be invariant across men and women.

#### 2.2.3. Statistical analysis

Participant characteristics based on sex were assessed via independent samples *T*-test and Chi-squared testing. The intercorrelations of the study variables were examined using Pearson’s Product Moment Correlation analysis. For all of the mentioned statistical methods, assumptions were evaluated before. In this study, descriptive statistics were presented as percentages “%.” Mean ± standard deviation (SD) was used to present the scores for the IES-scores and its subscales. Separate multiple linear regression analyses were conducted to determine if total IE and its subscale scores differed across sexes. It was also conducted to assess the relationship between IE and BMI across sexes. The regression models were adjusted for income as well as education except for EPR subscale which only adjusted for income due to its significant correlation with the study variables. Statistical significance was set at *p*-value < 0.05. All statistical analyses were performed using two-sided tests, carried out by SPSS software version 27 (IBM Corporation, Armonk, New York, United States). For the total IES-scores, RHSC, and BFCC regression models, a bootstrapping technique was implemented instead of the conventional computing techniques because the normality assumption was violated.

## 3. Results

A total of 360 participants who were 26.5 ± 6 years of age (57.5%, *n* = 207 women; and 42.5%, *n* = 153 men) were included in the analyses. Participants demographic characteristics, including age, education, and income, are presented in Table 1. Participants had a mean BMI of 24.4 ± 4.9 kg/m^2^. The mean BMI of women and men included in the study were 24.7 ± 5.3 and 24 ± 4.3 kg/m^2^, respectively. More than half of the participants (76.7%) had a Bachelor’s degree or higher education. Nearly half of the participants were classified in the lowest category of income (48%). A majority of participants (92.8%) were Saudis. No significant differences were observed between men and women in terms of the demographic data. Means and standard deviations for the Intuitive Eating Scale-2 (IES-2) total score and the four subscales by sex, are presented in Table 2.

**Table 1:** Demographic characteristics of the study population (*N* = 360; Women = 207; Men = 153).

Demographic characteristics	Mean (SD) or %
Age (Years)	26.5 (6)
Women	25.0 (5.2)
Men	28.5 (6.4)
BMI (kg/m^2^)	24.4 (4.9)
Women	24.7 (5.3)
Men	24 (4.3)
Education	
High school	
Women	54.5%
Men	45.5%
Diploma	
Women	13.8%
Men	86.2%
Bachelor or higher education	
Women	62.7%
Men	37.7%
Income (Saudi riyal)	
<5,000	48%
5,000–15,000	36%
16,000–30,000	13%
>30,000	3%
Nationality	
Saudi	92.8%
Women	54.8%
Men	45.2%
Non-Saudi	7.2%
Women	92.3%
Men	7.7%

**Table 2:** Means and standard deviations for the Intuitive Eating Scale-2 (IES-2) total score and the four subscales by sex.

Scale	Women (*n* = 207)	Men (*n* = 153)
IES-total	2.90 (0.50)	2.79 (0.46)
UPE	2.87 (0.69)	3.02 (0.65)
EPR	3.18 (0.65)	3.16 (0.53)
RHSC	2.60 (0.90)	2.26 (0.92)
BFCC	2.81 (1.36)	2.38 (1.25)

IES-2, Intuitive Eating Scale-2; UPE, Unconditional permission to eat; EPR, Eating for physical rather than emotional reasons; RHSC, Reliance on hunger and satiety cues; B-FCC, Body-food choice congruence; n, number of participants.

**Figure 1:**
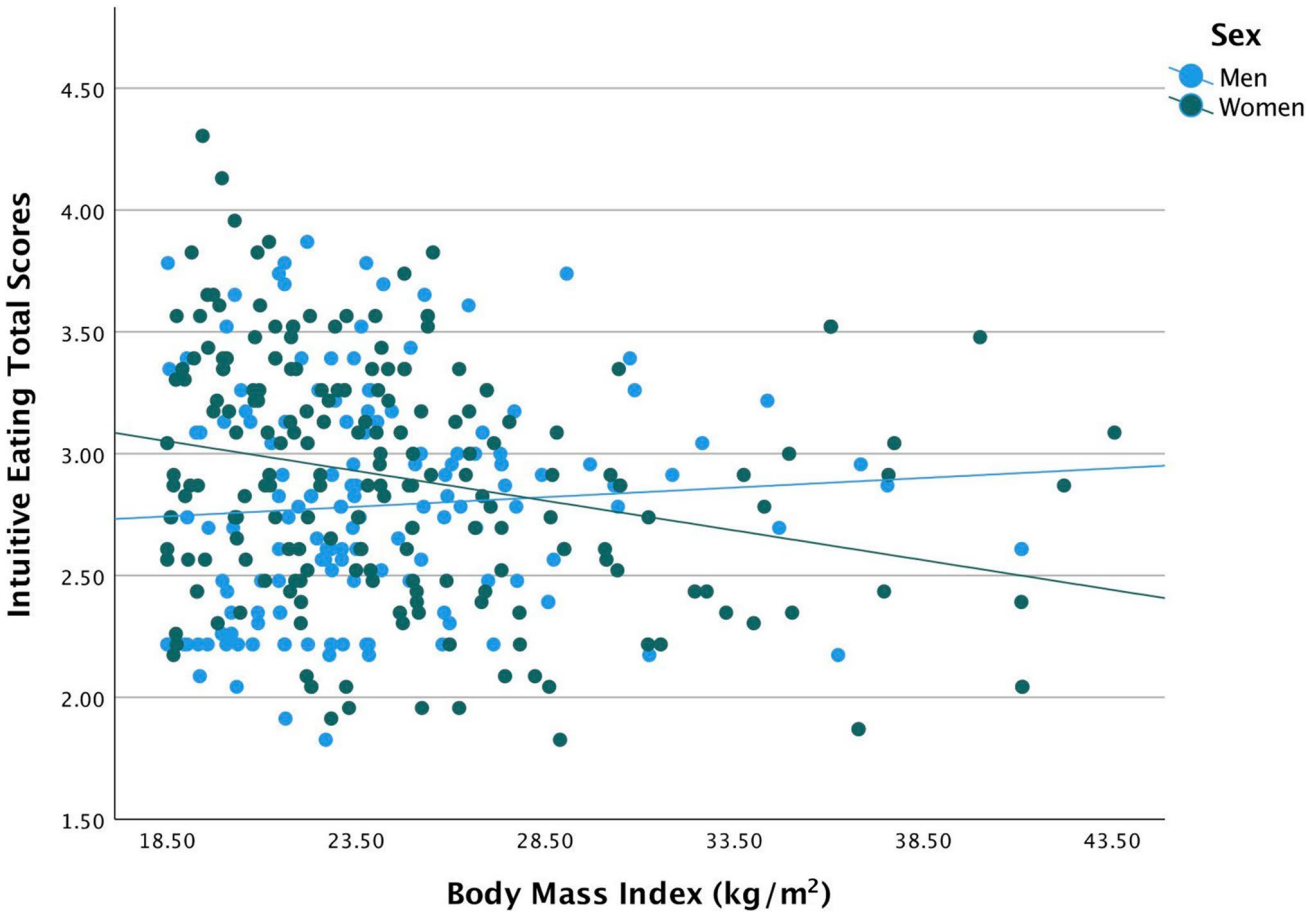
Indicates a negative association between total IE scores and BMI among women.

Results for the multiple linear regression analyses are shown in Table 3. For the total IE scores, there was a significant effect of sex, such that intuitive eating in women was greater than men [β (standardized beta coefficient) = 0.73, 95% CI: 0.22, 1.32, *p* = 0.01]. The model for the total IE score revealed a significant interaction between sex and BMI on IES after controlling for education, and income (*β* = −0.02, 95% CI: −0.05, −0.01, *p* = 0.01). This significant negative association means when the individual is female, the total IE score decreased by 0.02 for every unit increase in BMI. Figure 1 shows the association between total IE scores and BMI between sex.

**Table 3:** Multiple regression analysis assessing the association between intuitive eating scores and body mass index (BMI)^a^.

Scale	Variable	*β*	SE_b_	*P*-value	95% CI
Total IE scores^b^					
	(Constant)	2.82	0.32	<0.001	2.13, 3.43
	Sex	0.73	0.28	0.013*	0.22, 1.32
	BMI	0.004	0.01	0.60	−0.01, 0.02
	Interaction	−0.02	0.01	0.012**	−0.05, −0.01
UPE subscale					
	(Constant)	3.55	0.37	<0.001	2.82, 4.30
	Sex	0.53	0.39	0.17	−0.24, 1.30
	BMI	−0.01	0.01	0.28	−0.04, 0.01
	Interaction	−0.02	0.01	0.07	−0.06, 0.003
EPR subscale					
	(Constant)	3.26	0.32	<0.001	2.62, 3.62
	Sex	0.83	0.34	0.01*	0.15, 1.52
	BMI	0.004	0.01	0.70	−0.02, 0.03
	Interaction	−0.03	0.01	0.01**	−0.61, −0.01
RHSC subscale^b^					
	(Constant)	2.20	0.56	<0.001	1.15, 3.33
	Sex	0.13	0.51	0.80	−0.87, 1.13
	BMI	0.01	0.01	0.44	−0.01, 0.04
	Interaction	0.01	0.02	0.74	−0.03, 0.04
BFCC subscale^b^					
	(Constant)	1.34	0.76	0.06	−0.22, 2.81
	Sex	2.44	0.73	<0.001*	0.99, 3.94
	BMI	0.03	0.02	0.17	−0.01, 0.07
	Interaction	−0.08	0.02	0.003**	−0.14, −0.03

IE, Intuitive Eating; BMI, Body mass index; UPE, Unconditional permission to eat; EPR, Eating for physical rather than emotional reasons; RHSC, Reliance on hunger and satiety cues; B-FCC, Body-food choice congruence; β, unstandardized beta coefficient; SE_b_, SE for the unstandardized β; CI, confidence interval.

^a^Income and education were controlled in the model for total IES-2 score, UPE, EPR, RHSC, B-FCC except for EPR, which controlled for income only.

^b^Bootstrapped for coefficient.

*Statistically significant effect of sex on total IE score, EPR and, B-FCC subscale, *p* = 0.013, *p* = 0.017, *p* < 0.001, respectively.

**Statistically significant negative association between sex and BMI on total IE score, EPR and, B-FCC subscale, *p* = 0.0128, *p* = 0.016, *p* = 0.003, respectively.

Regarding UPE subscale, sex has no significant effect (*β* = 0.53; 95% CI: −0.2, 1.30; *p* = 0.17). The model did not display a significant interaction between sex and BMI on UPE after controlling for education, and income (*β* = −0.02, *p* = 0.07). Moreover, for EPR subscale, there was a significant effect of sex, such that EPR score in women was greater than men (*β* = 0.83, 95% CI: 0.15, 1.52, *p* = 0.01). The model for the EPR subscale revealed a significant interaction between sex and BMI on EPR subscale after controlling for income (*β* = −0.03, 95% CI: −0.02, 0.03, *p* = 0.01). This significant negative association means when the individual is female, the EPR score decreased by 0.03 for every unite increase in BMI.

Additionally, for RHSC subscale, sex has no significant effect (*β* = 0.13; 95% CI: −0.87, 1.13; *p* = 0.80). The model did not display a significant interaction between sex and BMI on RHSC after controlling for education, and income (*β* = 0.01, *p* = 0.74). Furthermore, for B-FCC subscale, there was a significant effect of sex, such that B-FCC score in women was greater than men (*β* = 2.44, 95% CI: 0.99, 3.94, *p* < 0.001). The model for the B-FCC subscale revealed a significant interaction between sex and BMI on B-FCC subscale after controlling for education, and income (*β* = −0.08, 95% CI: −0.14, −0.03, *p* = 0.003). This significant negative association means when the individual is female, the B-FCC score decreased by 0.08 for every unite increase in BMI.

## 4. Discussion

The present study examined the differences in IE and its subscales across sexes (women vs. men) in Saudi Arabia. In addition, the study assessed the relationship between IE and its subscales with body indices, specifically BMI, across sexes. The study findings indicated that women had significantly higher levels of IE compared to men. Results also suggested that women scored significantly higher on two of the IE subscales [Body-food choice congruence (BFCC) and Eating for physical rather than emotional reasons (EPR)] compared to men. The study also revealed a significant negative relationship between BMI and BFCC as well as EPR only in women.

Findings on IE across sexes have been inconsistent. Previous studies have reported equal level of IE, or one sex with a higher score than the other, mostly men (12, 15, 26, 29). On the contrary, our study revealed a significant sex difference in the total IE score, with higher scores in women than men. A possible explanation for this may be the fact that our sample excluded individuals who were current dieters. The researchers noted that IE was significantly higher among individuals who were never dieters compared to those who were former as well as current dieters (15). The author speculated that dieting might disrupt the innate ability of an individual to take notice of the body signals that regulate food intake. This disturbance may in turn affect individuals’ ability to differentiate physical from emotional hunger. Presumably, this may not apply to our women sample as they not only ate more intuitively but they also eat in response to physical needs rather than emotional ones compared to men. Therefore, studies should consider diet-related behaviors and their influence on food regulation signals when examining IE.

This inconsistency regarding study findings could also be attributed to the existence of other eating behaviors, such as restraint vs. emotional eating. In line with a previous study, researchers reported that women had lower IE than men. In addition, they also reported that these women had higher levels of restraint as well as emotional eating (29). These eating patterns rely on external guidance and predetermined rules that dictate eating decisions, whereas IE involves internal guidance and a willingness to eat when the body is in need (29). In our sample, these could be reflected in women reporting higher eating for physical rather than emotional reasons and body-food choice congruence subscales, and their BMI association with the subscales compared to men. However, these women may or may not practice restraint overeating, but, according to the EPR scores, they attenuated overeating in response to emotional triggers. One may assume that inner tuning, connecting, and listening to the body needs, may increase women’s senses toward hunger and satiety signals, thus, an appropriately response to triggers and a healthier body-food relationship. As mentioned, the sample of this study ruled out dieters or eating disorders but did not include an objective assessment of other eating behaviors that were not within the scope of the study. We assumed that the women in our sample did not follow extensive restrictive eating behaviors which could have resulted in a high IE score. Therefore, adding objective measures of other eating behaviors which may interfere with IE is worth considering.

Interestingly, the correlational findings of the current study were consistent with other studies, which reported an inverse relationship between IE and BMI (12, 14, 15, 30, 31). Our findings observed a conflicting directional correlation in women and men, contrary to some studies which reported similar relationships in both sexes. Consistent with our study, researchers reported the same inverse relationship in older women (32). Similar findings were noted in another study with younger women, which reported that BMI decreased with an increase in IE scores (14). In accordance with these studies, our findings may suggest that women who scored high on IE tended to have lower BMI while men scored high on IE tended to have high BMI. It is worth noting that the average BMI for women and men in this study was similar compared to other studies (14, 33). We hypothesize that women with normal BMI may be at minimum risk for the sociocultural pressure of thinness and dieting; thus, they might be more aware of and in harmony with their emotions and physical need for food (29, 34). This hypothesis could be supported by the negative correlation between BFCC, EPR and BMI in women as observed by other studies (19, 30). Another possible explanation might be related to the freedom from preoccupying food thoughts and the pressure of dieting that allows oneself to pay attention to the body’s needs, which promotes a healthier food-mind–body connection and, thus, a healthier BMI. This may suggest that eating for non-emotional reasons where food choices in agreement with body needs are the main contributors in the overall relationship between IE and BMI. This also suggests that the link between IE and BMI becomes more pronounced in women with low BMI. These data suggest that IE principles (BFCC and EPR) might be relevant for weight management in women but not in men in Saudi Arabia. In addition, IE and its subscales could be used as a screening tool to identify women who are likely to eat in response to emotional triggers and may be less likely to choose foods that are congruent with their body’s needs and preferences. Further studies are required to prove these hypotheses.

Important factors that might play a role in the observed discrepancies between our results and those of previous studies are the cross-cultural differences in foods and the mentality toward dieting. To the best of our knowledge, this is the first study to examine IE and its subscales across sexes and its relationship with BMI in the Saudi Arabian population. Eating styles and practices have been suggested to differ across cultures. In addition, reciprocal interactions between sex and sociocultural factors are known to influence food intake and choice (35). Thus, more studies should be conducted to identify the influence of cross-cultural differences on IE.

Our findings have several important clinical implications. The findings from this cross-sectional survey suggest that women eat more intuitively when they have a lower BMI whereas men eat less intuitively with a higher BMI. In addition, women with lower BMI tend to eat according to physical reasons rather than non-emotional reasons where food choices are in agreement with their body needs. The link between this non-restrictive eating approach and body mass may suggest the use of this approach to promote and maintain a healthy weight in this specific sample. Therefore, to encourage healthier weight-related outcomes, dietitians in Saudi Arabia should discuss the concept of IE with their patients, especially women. In addition, the findings may encourage facing the obesity dilemma differently by adopting an approach that focuses on the mentality of “body wisdom” instead of dieting itself or may necessitate the combination of both approaches.

The current study had a few limitations. First, it is worth noting that the distributed survey used the English version of IES-2 due to the unavailability of the Arabic version. Considering that Arabic is the native language of Saudi Arabia, the study sample may not be fully reflective of the general population. Second, the underrepresentation of the general population may limit the generalizability of the results. A representative sample would include Arabic speakers as well as more non-Saudis as they represent 41.6% of the population in Saudi Arabia (36). In addition, it would also include participants from different educational levels, such as middle and high school graduates as they represent 22.6 and 26.6% of the population; respectively. More individuals with an income between 5,000 and 15,000 would also make the sample more representative (37). Third, the use of a cross-sectional design limits the development of cause-and-effect relationships (38). Fourth, we recruited a convenience sample that led to half of the sample being within normal BMI in both groups, thus resulting in an underrepresentation of overweight and obese people. Despite these limitations, this study had several strengths that need to be mentioned. First, to the best of our knowledge, this study is the first to consider sex differences in the context of IE and its relation to BMI in Saudi Arabian population. This is an important first step in understanding how IE varies across sexes and how it is related to body status in this understudied population. Second, the study had a relatively large sample size, with diverse age ranges and educational levels.

The findings from the present study are promising and suggest the need for future research on this topic. To the best of our knowledge, no adaptation of the scale has been conducted in the Saudi population; therefore, this study is novel as it highlights the potential value of translating and validating the IES-2 in Arabic. Additionally, the cross-sectional design of the current study does not establish causality; therefore, it was ambivalent whether lower BMI resulted in an increase in IE or whether having the ability to eat intuitively lead to a normal BMI. Further evidence is needed to investigate the potential association between self-regulation and BMI in the general population. Additionally, our findings indicate the necessity of evaluating IE in a larger sample with different BMI categories and eating styles across sexes. Future research should also use longitudinal study designs which would aid in tracking individuals’ weight changes and its impact on levels of IE. Exploring IE practices and its relationship with BMI could reveal possible mediating effects of eating styles in the Saudi Arabian population. Future studies should attempt to replicate the sex disparities discovered in this study with a sample of dieters vs. non-dieters. The significant inverse association between IE and BMI warrants the need for intervention studies that would examine the effect of IE training on weight status in women with higher BMI.

## 5. Conclusion

This study revealed that women eat more intuitively, rely on physical hunger cues rather than emotional triggers, and their food choices are related to their body’s needs compared to men. Women’s BMI was inversely associated with total IES, BFCC, and EPR while in men, BMI was positively associated with total IES. This suggests that IE and some of its principles may be a protective strategy against weight gain at a certain BMI in the women population in Saudi Arabia. The current findings highlight the importance of translating and validating the IES in the Arabic language, to better represent and understand Saudi culture. Prospective studies in the Saudi population are required to investigate the temporal and causal relationships between IE and BMI. Taken together, the current and previous findings should encourage dietitians in Saudi Arabia to discuss the concept of IE and its components (BFCC and EPR) with their patients, especially women as a helpful tool in weight management practice to promote healthier outcomes.

## Data availability statement

The raw data supporting the conclusions of this article will be made available by the authors, without undue reservation.

## Ethics statement

The studies involving human participants were reviewed and approved by the Ethical Committee of the Faculty of Applied Medical Sciences, King Abdulaziz University (FAMS-EC2020-0016). The patients/participants provided their written informed consent to participate in this study.

## Author contributions

EA and MN conceived the study, conducted part of the analyses, interpreted the results, and wrote the manuscript. All authors critically reviewed the manuscript and approved the final version submitted for publication.

## Conflict of interest

The authors declare that the research was conducted in the absence of any commercial or financial relationships that could be construed as a potential conflict of interest.

## Publisher’s note

All claims expressed in this article are solely those of the authors and do not necessarily represent those of their affiliated organizations, or those of the publisher, the editors and the reviewers. Any product that may be evaluated in this article, or claim that may be made by its manufacturer, is not guaranteed or endorsed by the publisher.
